# Low-rank human-like agents are trusted more and blamed less in human-autonomy teaming

**DOI:** 10.3389/frai.2024.1273350

**Published:** 2024-04-29

**Authors:** Jody Gall, Christopher J. Stanton

**Affiliations:** The MARCS Institute for Brain Behaviour and Development, Western Sydney University, Parramatta, NSW, Australia

**Keywords:** human-autonomy teaming, trust, blame, anthropomorphism, status, power distance orientation, shared tasks

## Abstract

If humans are to team with artificial teammates, factors that influence trust and shared accountability must be considered when designing agents. This study investigates the influence of anthropomorphism, rank, decision cost, and task difficulty on trust in human-autonomous teams (HAT) and how blame is apportioned if shared tasks fail. Participants (*N* = 31) completed repeated trials with an artificial teammate using a low-fidelity variation of an air-traffic control game. We manipulated anthropomorphism (human-like or machine-like), military rank of artificial teammates using three-star (superiors), two-star (peers), or one-star (subordinate) agents, the perceived payload of vehicles with people or supplies onboard, and task difficulty with easy or hard missions using a within-subject design. A behavioural measure of trust was inferred when participants accepted agent recommendations, and a measure of no trust when recommendations were rejected or ignored. We analysed the data for trust using binomial logistic regression. After each trial, blame was apportioned using a 2-item scale and analysed using a one-way repeated measures ANOVA. A post-experiment questionnaire obtained participants’ power distance orientation using a seven-item scale. Possible power-related effects on trust and blame apportioning are discussed. Our findings suggest that artificial agents with higher levels of anthropomorphism and lower levels of rank increased trust and shared accountability, with human team members accepting more blame for team failures.

## 1 Introduction

McNeese et al. (2018) predict that autonomous agents could replace human teammates in various roles, including pilots, navigators, or photographers who conduct surveillance whilst negotiating enemy activity. Human-Autonomy Teaming (HAT) describes teams comprised of at least one human and one autonomous agent, both recognised as fulfilling a distinct role on the team and striving to achieve an objective (O’Neill et al., 2020). As autonomous agents increase in Artificial Intelligence (AI), they have become increasingly agentic, proactive, and synchronised with humans that government, industry, and the military are trialling HAT in real-world scenarios (O’Neill et al., 2020). Examples include smart city management (Allam and Dhunny, 2019), health care (Wilson and Daugherty, 2018), and cyber defence (Morgan et al., 2020).

It has been argued that HAT could improve team performance compared to teams comprised only of people in situations of uncertainty (Cummings, 2014). It may be critical in missions without predetermined boundaries, such as warfare (Chen and Barnes, 2014). However, for humans to complete real-world missions with autonomous agents as part of human-autonomous teams, factors influencing trust and teamwork in AI teammates must be considered when designing autonomous agents. Furthermore, when missions fail, policies for shared accountability will be needed to apportion blame for shared task failures.

### 1.1 Blame

According to Lyons et al. (2021) recent review of the HAT literature, there is a notable research gap on shared accountability in HAT. A key finding is that asymmetries are often found when comparing consequences and expectations of human-based actions and decisions with machine ones, with robots given less blame for failure than humans. For example, in a study by Li et al. (2016), participants judged an autonomous vehicle less responsible for a traffic accident of equal severity than a human driver. Contrary to these findings, Liu and Du (2021) found that people judge an automation-caused crash more harshly, impute more responsibility and blame to automation and designers, and believe victims deserve more compensation than a crash of equal severity caused by humans.

Malle et al. (2019) offer another example of blame asymmetry. Participants answered two moral judgment questions after reading a narrative that presented a human drone pilot, an autonomous drone, and an AI agent facing an ethical dilemma in a military context. First, would the decision be morally wrong to cancel a strike and save a child but risk a terrorist attack? Or, launch a strike to eliminate a terrorist attack but risk a child’s life? Second, how much blame would they deserve for their decision? Results of the study showed that the human pilot received far more responsibility for cancelling the strike than for launching the strike. However, the artificial agents received similar blame for cancelling and launching. Malle et al. (2019) suggest people are less inclined to see AI agents embedded in social structures and, as a result, apportion blame differently than humans.

Interestingly, 72 % of participants reported feeling comfortable making moral judgments of wrongness and blame to an autonomous agent facing an ethical dilemma; however, only 51 % felt comfortable blaming an autonomous drone. The researchers suggest that drones may conjure an image of a passive metal device, whereas AI’s better fit the model of an agent that deserves to be praised and blamed for their actions. The researcher’s assumptions are justified by recent findings surrounding CASA (Computers are Social Actors) (Nass et al., 1994).

### 1.2 Blame, anthropomorphism, and rank

According to the Computers are Social Actors (CASA) paradigm (Nass et al., 1994), humans apply social rules to their computer interactions. One factor influencing CASA effects is anthropomorphism, the perception of human characteristics in an entity indicating its potential for social interaction (Gambino et al., 2020). In their recent review of the CASA literature, Gambino et al. (2020) found anthropomorphism to be a crucial determinant in how agents are evaluated, with higher levels of anthropomorphism more likely to produce CASA effects.

Assuming that interactions between people and humanoid robots follow the rules of human-human exchanges, Lei and Rau (2021) hypothesised that participants teamed with an autonomous robot named Nao (Aldebaran, 2022) would attribute responsibility for successes and failures between themselves and Nao in a self-serving manner. Further, the self-serving bias would increase as the robot’s status increased from subordinate peer to supervisor. The results suggest that when paired with a robot subordinate, peer, and supervisor of a joint task, humans attribute more responsibility to themselves for success, supporting the researcher’s hypothesis of a self-serving bias. However, when attributing responsibility for failure, participants attributed more responsibility to themselves when paired with robot subordinates and peers but more to robot supervisors. The results for robot supervisors showed similar patterns found by Hinds et al. (2004), who compared responsibility, attribution of credit and blame between three levels of status, subordinate, peer, and supervisor, and three levels of partner, human, human-like robot, and a machine-like robot. Again, the findings suggest participants felt more responsibility when paired with robot subordinates and peers than supervisors and blamed robot supervisors significantly more.

In human-human interactions, Willemsen et al. (2018) argue that social roles and hierarchies, such as supervisor and subordinate, can significantly affect the attribution of praise and blame. For example, in a recent study using a human-human vignette by Kaspar et al. (2016) comparing attribution of praise and blame between a boss and employee jointly implementing a company strategy, the boss received significantly more blame for the outcome than the employee.

From the findings of Gambino et al. (2020), we argue that higher levels of anthropomorphism will invoke CASA effects and expect to see similar findings as human-human interactions (see Kaspar et al., 2016), with more blame apportioned to high-rank human-like teammates than low-rank human-like teammates (H1). We also expect more blame attributed to high-rank human-like teammates than high-rank machine-like but less blame to equal-rank and low-rank human-like teammates than equal-rank and low-rank machine-like (H2).

### 1.3 Trust

In a review and analysis of 24 studies that measured trust in HAT (O’Neill et al., 2020), higher levels of agent autonomy (Azhar and Sklar, 2017), and higher levels of transparency (Boyce et al., 2015; Sharifheravi et al., 2020), had a positive effect on trust. When an autonomous agent’s personality (Hanna and Richards, 2018), values (Mehrotra et al., 2021), and work style are similar to participants, they trust agents more, even preferring to work with an agent over a human in low-risk situations (You and Robert, 2018). Other factors influencing higher levels of trust in autonomous agents include experience with similar computers or computers that employ independent agents (Chen et al., 2011), team training (Walliser et al., 2019), and agent reputation (Hafizoglu and Sen, 2018). Factors that influence lower levels of trust in autonomous agents include lower reliability (Hafizoğlu and Sen, 2019; Sharifheravi et al., 2020) and high power distance orientation (Chien, 2016). However, O’Neill et al. (2020) suggest more research is needed on human individual difference variables that impact human-agent interaction to ascertain whether similarity or complementarity on such differences is optimal. For example, do participants trust autonomous agents that are anthropomorphic and human-like more than machine-like or agents with a similar status more than agents with a lower or higher status?

### 1.4 Trust, anthropomorphism, and rank

In a study by Sharifheravi et al. (2020) investigating anthropomorphism, the researchers used MAHVS (Multiple Autonomous Heterogeneous Vehicle Simulation) to measure the effects that high and low anthropomorphism had on task performance and trust toward an autonomous agent. MAHVS is a low-fidelity air traffic control simulation in which participants were teamed with an independent agent and tasked with guiding aircraft to avoid collisions and enemy fire, with recommendations accepted or rejected by the agent to infer a behavioural measure of trust. Results from the study suggest that an agent with higher levels of anthropomorphism had no significant effect on trust compared to a lower-anthropomorphic system, contrary to the researcher’s hypothesis and previous findings by Calhoun et al. (2019). A limitation of the study suggested by the researchers was that perhaps the human-like avatar was not anthropomorphic enough to confirm their hypothesis.

Christoforakos et al. (2021) investigated the influence of anthropomorphism on the interrelations of competence and warmth on trust in two studies. Participants watched a video of a human playing a shell game with either a low-anthropomorphic robot or a high-anthropomorphic robot with low or high competence in study one and low or high warmth in study two. The results suggest competence and warmth positively affected trust, supporting the researcher’s hypothesis and prior research on the transferability of interpersonal trust in human-computer interactions (Kulms and Kopp, 2018). However, the prediction that higher levels of anthropomorphism would moderate the role of competence and warmth on trust in human-robot interactions was unsupported, with the researchers suggesting the relatively weak manipulation of anthropomorphism as a contributing factor.

de Visser et al. (2016) investigated the effect anthropomorphism has on trust formation, violation and trust repair in a pattern detection task performed by participants with the assistance of a computer (low-anthropomorphic), human agent (high-anthropomorphic) or an avatar considered a mean level of anthropomorphism between the two. The results showed that anthropomorphism decreased initial trust, consistent with automation bias (Dzindolet et al., 2003), where people ascribe higher initial trust to low-anthropomorphic agents. Further, higher levels of anthropomorphism reduced the impact of trust violations, with agents that appear or act human forming a higher resistance to changes in trust despite poor automation performance. In addition, anthropomorphism improved trust repair.

In a study investigating the effects of agent reputation’s influence on human trust in agent teammates, Hafizoglu and Sen (2018) paired participants with a disembodied agent in a game of trust. Participants were tasked with transcribing words from audio to text; with the number of words they transcribed, a critical decision was made based on their trust in the agent’s reputation to contribute to the team task. In addition, a cover story manipulated the importance of their teammate being trustworthy or untrustworthy. Results from the study suggest that a positive reputation led to significantly greater trust than a negative reputation.

In another study investigating trust and status by Beton et al. (2017), participants played a labyrinth game with either a robot leader or a robot follower as a teammate to compare the effect perceived safety and intelligence have on human trust in AI teammates. The results suggest that participants regard a robot teammate follower as more competent, knowledgeable, and trustworthy than a leader in a maze game.

In a military context, however, a positive reputation is often conferred on higher-ranked individuals because of the authority granted to these leaders, with increased trust influenced by such categorisation (Adams and Webb, 2002). For example, recommendations are trusted more from superiors than subordinates. According to Adams and Webb (2002), such category-based trust can emerge before developing person-based trust based on whether a person is predictable and dependable.

If higher levels of anthropomorphism invoke CASA effects (Gambino et al., 2020), prompting humans to confer category-based trust to high-rank human-like teammates similar to high-rank human teammates in the military (Adams and Webb, 2002), we would expect more recommendations accepted from high-rank human-like teammates than high-rank machine-like (H3). We would expect further evidence with more recommendations accepted from high-rank human-like teammates than low-rank human-like (H4) and no significant difference in recommendations accepted from high-rank machine-like teammates than low-rank machine-like (H5).

### 1.5 Present study

Our research is informed by the CASA paradigm (Nass et al., 1994). This theoretical framework states that humans apply social rules to their interactions with computers similarly to human-human interactions. Our first objective is to investigate whether higher levels of anthropomorphism invoke CASA effects (Gambino et al., 2020). Our second objective is to investigate power distance orientation’s effect on users’ trust in autonomous agents and blame apportioning. Finally, our third objective is to ascertain whether the similarity or complementarity of human difference variables that impact trust and blame apportioning is optimal.

During shared tasks in air traffic control with varying levels of difficulty and decision costs, our study will compare blame apportioned between participants and an artificial agent represented by a machine-like avatar (low-anthropomorphic) and an artificial agent designated by a human-like avatar (high-anthropomorphic). Further, a comparison of accepted agent recommendations is used to measure trust and no trust when recommendations are rejected, ignored, or when participants take manual control of the vehicles. Finally, to invoke power-related effects, we introduce agent rank, manipulated as either subordinate, peer, or superior to participants.

In conducting our research, we hope to interest the following stakeholders. First, policymakers. When shared tasks fail in HAT, policies will be needed to apportion blame between human and autonomous team members; therefore, understanding artificial teammates’ design factors that influence human teammates’ sense of shared accountability is essential. Second, agent designers. Human factors influencing trust and shared accountability in HAT must be considered if humans are expected to complete shared tasks with autonomous agents—lastly, researchers. Suppose higher levels of anthropomorphism invoke CASA effects (Gambino et al., 2020). In that case, we hope to demonstrate that priming social roles and hierarchies prompt humans to apportion blame between superior and subordinate human-like teammates similarly to how they apportion blame to human employers and employees (Kaspar et al., 2016) and confer category-based trust to high-rank human-like teammates similar to high-rank individuals in the military (Adams and Webb, 2002).

## 2 Materials and methods

### 2.1 Participants

The study was approved by Western Sydney University’s Human Ethics Committee (H1480), and participants provided informed written consent. Thirty-two participants (48.4% male, 51.6% female; *M*_age_ = 35.06, SD = 9.95; age range = 20–56 years) were recruited via an online Facebook advertisement. Participation was voluntary, and participants received no financial reimbursement.

### 2.2 Task

**Figure 1:**
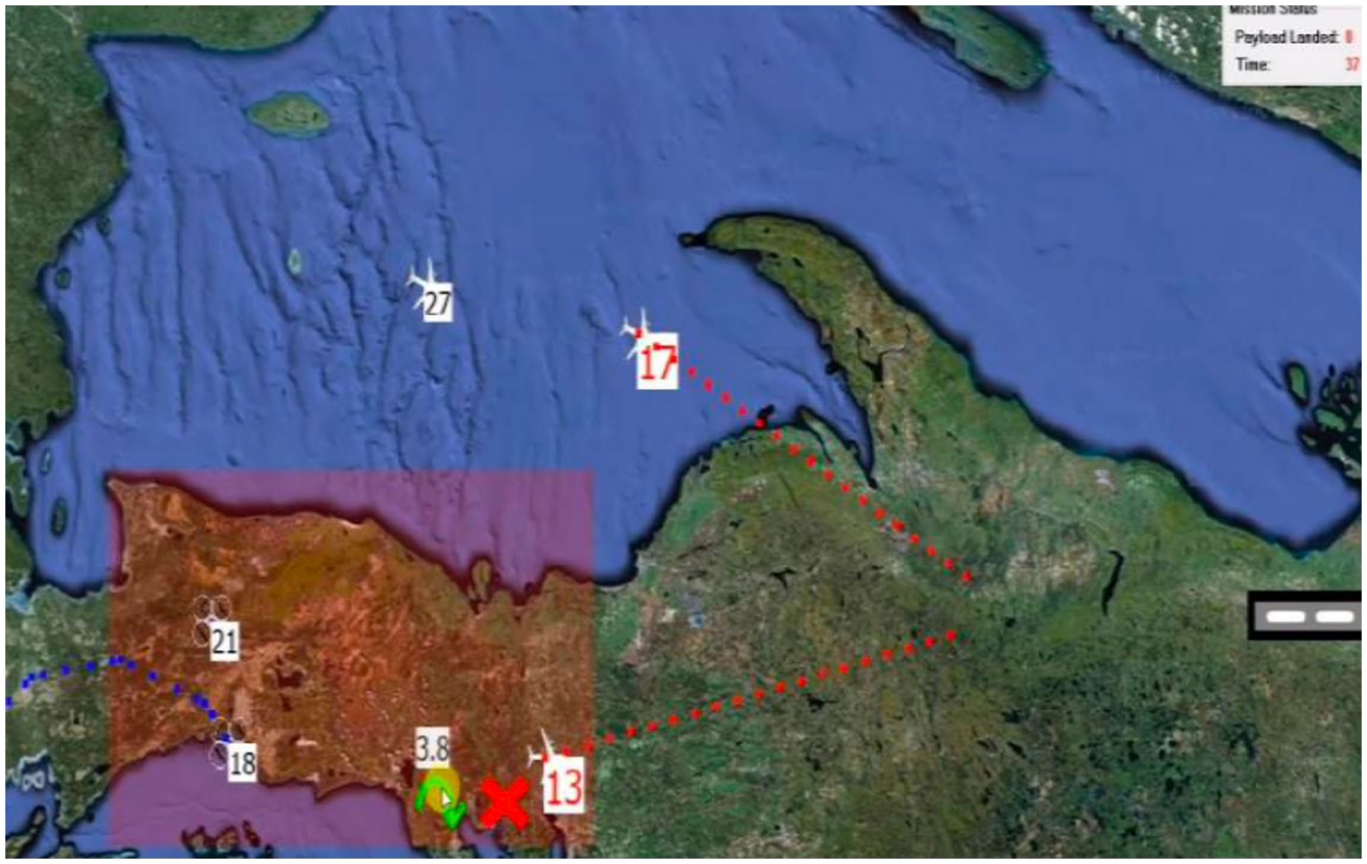
MAHVS (multiple autonomous heterogeneous vehicle simulation). The MAHVS simulation. Participants guide numerous aircraft to safety (landing strip at the right of the screen). Aircraft passing through the danger zone (indicated by the red rectangle) risk being destroyed by enemy fire. Green ticks or arrows indicate routing recommendations offered by the virtual agent over the aircraft. Participants can accept recommendations by clicking on the green tick/arrow, reject the recommendations by clicking on the red cross, or manually control the aircraft by clicking directly on an aircraft.

For this study, we use MAHVS (see Figure 1). MAHVS was developed to answer human factors research questions when a single operator manages multiple autonomous vehicles in real-time environments, such as human-on-the-loop decision-making, attention, and cognitive load. Furthermore, to improve operator performance, an AI system, acting as a teammate, offers recommendations in real-time, which can be either accepted, rejected, or ignored, with the decisions used to infer a behavioural measure of trust. A demonstration of MAHVS can be viewed at https://youtu.be/Yz6rfCr1FMw.

MAHVS is a highly parametrised system that allows the manipulation of variables such as task difficulty, cognitive load, time pressures, anthropomorphic features of the AI such as faces, voices, and names, as well as social processes through cover stories. Another variable that can be manipulated using MAHVS is the AI’s reliability and competence using false-positive and false-negative error rates. For example, all AI teammates made recommendations for the current study with a 10% false positive and 10% false negative error rate. Keeping error rates equal reduced the confounding effect that detecting unequal incorrect recommendations from teammates would have on participants’ trust and shared accountability with lower, equal, and higher-ranked AI teammates.

In MAHVS, each trial has a ‘task performance’ metric, calculated by the number of aircraft safely landing and the time to complete the mission. Aircraft value varied in the number of supplies carried by drones or passengers by passenger jets, with the total of all aircraft values for each trial equaling 100. For example, a passenger jet carrying 23 passengers had a value of 23, whilst a drone carrying 13 kg of supplies had a value of 13. In addition, each trial counts down from 40 s, with any remaining seconds of the mission added to the score.

### 2.3 Experimental design

This study applied a factorial 2 × 3 × 2 × 2 repeated-measures within-subjects design. The independent variables under investigation are teammate type (low/high anthropomorphism), teammate rank (lower, equal, higher), decision cost (supplies/human), and task difficulty (easy/hard). Dependent variables under investigation are blame apportioning, trust, and power distance orientation.

Each participant completed 24 trials, comprised of six blocks of trials with four trials in each block. Each block represented a combination of anthropomorphism and rank; thus, the six blocks consisted of human-like lower-rank, human-like equal-rank, human-like higher ranked, machine-like lower-rank, machine-like equal-rank, and machine-like higher-ranked. The four trials within each block manipulated task difficulty (easy versus hard) and decision cost (human versus supplies). The block order and trial order were counterbalanced to prevent order effects.

Hypothesis 1 and 2 surrounding blame apportioning will be tested using one-way repeated measures ANOVA with Sidak *post hoc* comparisons. Hypothesis 3, 4, and 5 surrounding trust will be tested using binomial logistic regression.

**Figure 2:**
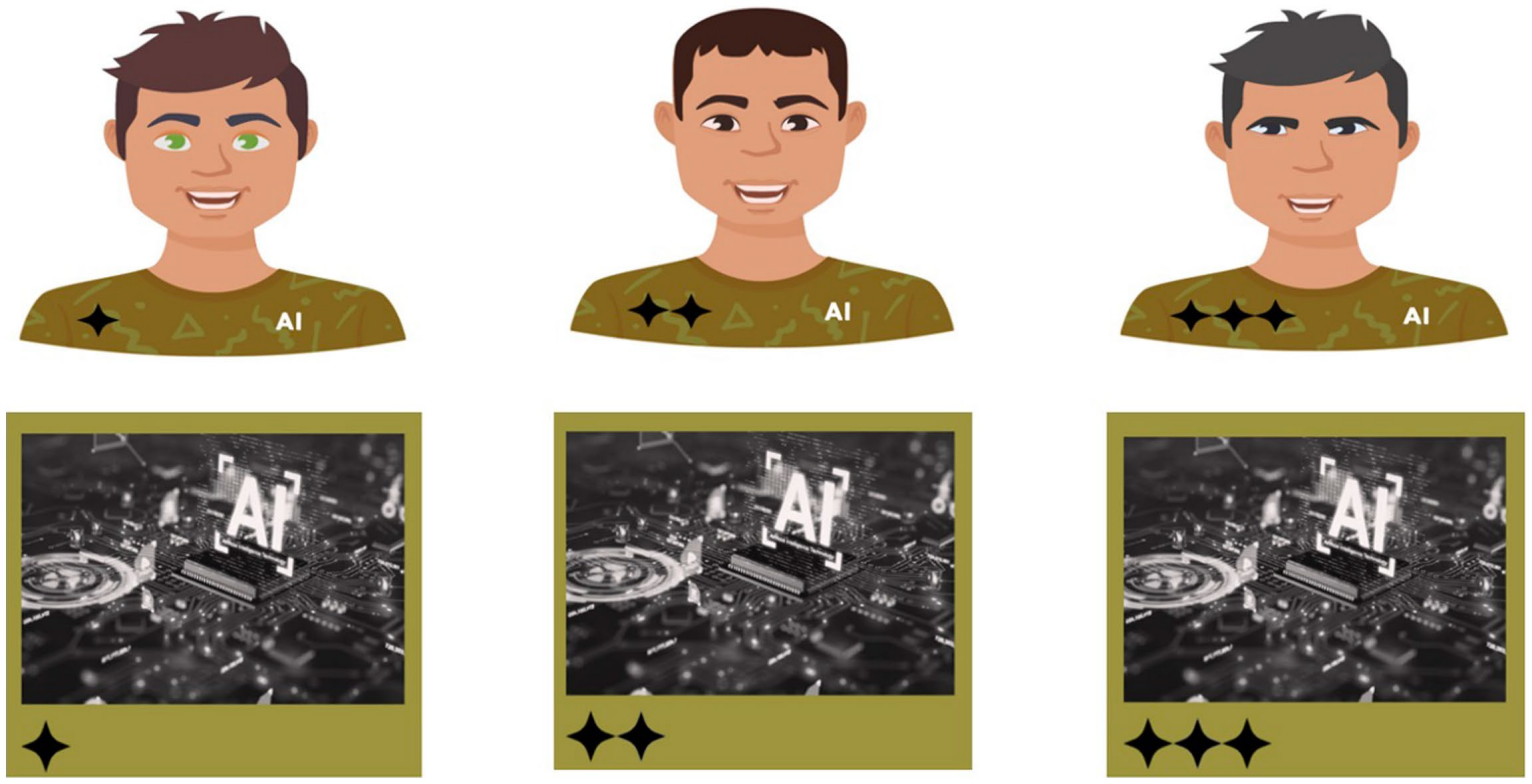
Participants’ six different teammates offered recommendations during MAHVS. High-anthropomorphic agents’ top three avatars and low-anthropomorphic agents’ bottom three avatars. Rank displayed from left to right: One-star subordinate, two-star peer, and three-star superior.

When a teammate’s anthropomorphism was high, it had a human-like appearance and embodiment (see Figure 2, top three avatars). We used Adobe Express to create three male avatars with slight differences in appearance, such as eye colour, hair colour and hairstyles. The avatar’s army green shirt displayed rank as one, two, or three stars. We used army green to stimulate a military context. AI was embedded on the shirt to reduce the confounding effect that may arise if participants forget their teammate is artificial intelligence and not human. When a teammate’s anthropomorphism was low, it was designated by a machine-like avatar (see Figure 2, bottom three avatars). We used Adobe Express to create three avatars with identical appearances other than rank, which was displayed as one, two, or three stars. Again, we used army green to stimulate a military context.

Decision cost was manipulated by the aircraft type, either passenger jets or drones. Participants were told passenger jets carried people, and drones carried supplies. Hence, participants are required to make decisions to maximise their scores whilst minimising their decision costs. For example, a passenger jet carrying 23 passengers and a drone carrying 23 kg of supplies passing over the ‘danger zone’ have the same numeric value of 23. However, the decision cost of a shot-down passenger jet is 23 lives hypothetically, whilst the drone is 23 kg of supplies.

Task difficulty was manipulated by the number of aircraft or drones, with low difficulty condition having four drones and the high difficulty condition having up to 8 drones. Rank was manipulated by the number of stars the AI teammate had, namely one, two or three stars.

### 2.4 Procedure

The MAHVS air traffic control game was explained to participants. After, participants completed two practice trials. After completing two practice trials, participants were assigned a rank of two stars regardless of performance during the trial. Keeping a constant rank for participants allowed us to analyse the effect pairing participants with lower, equal, or higher-rank teammates had on blame apportioning and trust.

Participants then completed 24 trials in which they were responsible for guiding numerous aircraft to safety whilst avoiding the “danger zone” and collisions with other aircraft. Individual trials varied in the type of aircraft (drones carrying supplies or jets carrying passengers), teammate type (low/high anthropomorphism), teammate rank (lower, same, higher), and task difficulty (easy/hard). The experiment’s duration was 30 min.

Before each trial, participants learned which teammate type they were paired with as the avatars were visible. For each trial with high-anthropomorphic teammates, participants were greeted with “I am here to help the mission succeed,” recorded in an English Australian accent using Microsoft 365 read-aloud function. Using text-to-voice increased the level of anthropomorphism of the high-anthropomorphic agents. In contrast, the low anthropomorphic teammates remained silent. During a trial, the avatars were not visible; however, at the end of each trial, a screen appeared with the participant’s teammate and a summary of the team’s performance, displaying a score out of 100 (based upon the number of aircraft safely landed and time taken). Throughout each trial, a behavioural measure of trust was secured by MAHVS when participants accepted agent recommendations and a measure of no trust when participants rejected or ignored agent recommendations or took manual control of the vehicles. After each trial, the participants blamed their teammates for their team’s performance using two items on a seven-point Likert scale, one representing less and seven representing more (see Table 1). We adopted the attribution of blame scale from Hinds et al.’s (2004) study, which investigated the effects of robot appearance and status on human-robot collaboration.

**Table 1:** Items used to measure participant’s attribution of blame after each trial.

Scales and items
Attribution of Blame
I hold my partner responsible for any errors that we made on this task.
My partner is to blame for most of the problems we encountered in accomplishing this task.

After each trial, participants answered, “Do you think your teammate was a leader or a follower in the missions?” on a seven-point Likert scale, with one representing a follower and seven representing a leader. We adapted this question from Lei and Rau’s (2021) study into the effect of relative status on responsibility attributions in human-robot collaboration to check the effectiveness of our status manipulation.

After completing all trials, participants completed a paper-based post-experiment questionnaire with a seven-item scale measuring the human likeness of all six teammates (see Table 2).

**Table 2:** Items used to measure participant’s perception of teammate’s human likeness.

Scale and items
Human likeness
To what extent do the robots
Have human-like attributes?
Look like a machine or mechanical device? ª
Have characteristics that you would expect of a human?
Look like a person?
Have machine-like attributes? ª
Act like a person?
Act like a machine? ª

ª Item was reverse scored.

The seven items were measured on a seven-point Likert scale, with one representing less and seven representing more. We adopted this scale from Hinds et al. (2004) to check whether manipulating anthropomorphism for high-anthropomorphic teammates was effective. Further, a seven-item scale measuring power distance orientation was included (see Table 3). The power distance orientation scale was adopted from Lei and Rau (2021). The items were scored on a seven-point Likert scale from one strongly disagree to seven strongly agree. We measured the power distance orientation of participants to examine any power-related effects on trust and blame apportioning.

**Table 3:** Items used to measure participant’s power distance orientation.

Scale and items
Power distance orientation
In most situations, managers should make decisions without consulting their subordinates.
Once a top-level executive makes a decision, people working for the company should not question it.
In work-related matters, managers have the right to expect obedience from their subordinates.
Employees should not express disagreement to their managers.
Managers should be able to make the right decisions without consulting others.
Employees should not disagree with management decisions.
It is better for people to not question the decisions of those in authority.

Furthermore, a series of open-ended questions were incorporated to gather additional qualitative data that could assist in interpreting the reasons participants trust and blame artificial teammates. The four questions integrated were “Which teammate did you trust/blame the most and why?’ and ‘Which teammate did you trust/blame the least and why?”. Informed by an essentialist perspective, we took a deductive approach to our thematic analysis and focused on specific aspects of trust and blame. First, category-based, based on anthropomorphism and the status of artificial teammates. Second, person-based, based on the predictability and dependability of artificial teammates.

## 3 Results

### 3.1 Quantitative

All statistical analyses were performed using SPSS version 28. Data screening found evidence that participant 24 did not complete the experiment; therefore, Participant 24 was omitted from the study.

A seven-item Likert scale measured the participant’s perception of the agent’s human likeness between the three high-anthropomorphic agents and three low-anthropomorphic agents from one more human-like to seven less human-like. Reliability of the scale was updated to reflect the current study sample Cronbach’s 𝛼 = 0.79. A dependent sample T-Test showed a significant difference between high-anthropomorphic agents (*M* = 4.76, SD = 1.32) and low-anthropomorphic agents (*M* = 2.03, SD = 0.94), *t*(30) = 8.50, *p* < 0.001. Hence, the manipulation of anthropomorphism was effective.

To verify our rank manipulation, we measured participants’ perception of their teammate’s leadership quality after each mission using one item on a seven-point Likert scale from one follower to seven leaders. Means and standard deviations for artificial teammates can be found in Table 4. The results of an ANOVA using Greenhouse–Geisser correction for sphericity violation found no evidence to support the effectiveness of the manipulation *F*(3.67,110.93) = 0.21, *p* = 0.921, η_p_^2^ = 0.01. Therefore, any findings associated with rank should be interpreted with caution.

**Table 4:** Means and standard deviations from follower leader question.

	*M*	*N*	SD
High-rank human-like	3.89	31	1.06
Equal-rank human-like	3.75	31	1.10
Low-rank human-like	3.80	31	1.09
High-rank machine-like	3.89	31	1.39
Equal-rank machine-like	3.95	31	0.96
Low-rank machine-like	3.77	31	1.12

Power Distance Orientation was measured using a seven-item scale on a seven-point Likert scale from one, low power distance orientation, to seven, high power distance orientation. Reliability of the scale was updated to reflect the current study sample Cronbach’s 𝛼 = 0.68. The results revealed participants’ low power distance orientation (*M* = 2.91, SD = 0.96).

A two-item blame scale measured the participant’s attribution of blame to each of the six artificial teammates operating under four conditions. The conditions were decision cost (supplies or passengers) and task difficulty (easy or hard). Scores from both questions were averaged to create a reliable attribution of blame scale. Reliability of the scale was updated to reflect the current study sample Cronbach’s 𝛼 = 0.99. Means and standard deviations for artificial teammates operating under each condition can be found in Table 5.

**Table 5:** Means and standard deviations for blame apportioning by human likeness and rank.

Conditions								
	Supplies easy		Supplies hard		Passengers easy		Passengers hard	
Teammate	*M*	SD	*M*	SD	*M*	SD	*M*	SD
Human-like								
Low-rank	2.92	1.98	3.58	2.33	2.48	1.74	2.74	1.96
Equal-rank	3.05	2.00	3.77	2.02	2.35	1.65	3.84	1.84
High-rank	2.82	1.83	4.05	2.02	3.21	2.18	4.13	1.72
Machine-like								
Low-rank	2.74	1.62	3.87	1.75	2.35	1.71	3.92	2.00
Equal-rank	2.40	1.39	3.40	1.68	2.58	1.51	3.65	2.01
High-rank	2.50	2.00	4.02	1.87	3.03	1.89	3.19	1.85

One-way repeated measure ANOVA was used to test blame apportioning. After converting raw scores to *z* scores, there was no evidence of outliers at *z* = ±3.29. Shapiro–Wilk test of normality was significant, although, given the large sample size (*N* = 31), this has little effect on the accuracy of the results. Mauchly’s test of sphericity was met. Results from a one-way repeated measures ANOVA found evidence of a main effect for task difficulty on blame apportioning *F*(1,30) = 33.56, *p < 0*.001, η_p_^2^ = 0.53, a two-way interaction for anthropomorphism and rank, *F*(2,60) = 4.02, *p* = 0.023, η_p_^2^ = 0.12, a three-way interaction for anthropomorphism, rank, task difficulty *F*(2,60) = 4.51, *p* = 0.015, η_p_^2^ = 0.13, and a three-way interaction for rank, decision cost, and task difficulty *F*(2,60) = 4.07, *p* = 0.022, η_p_^2^ = 0.12.

**Figure 3:**
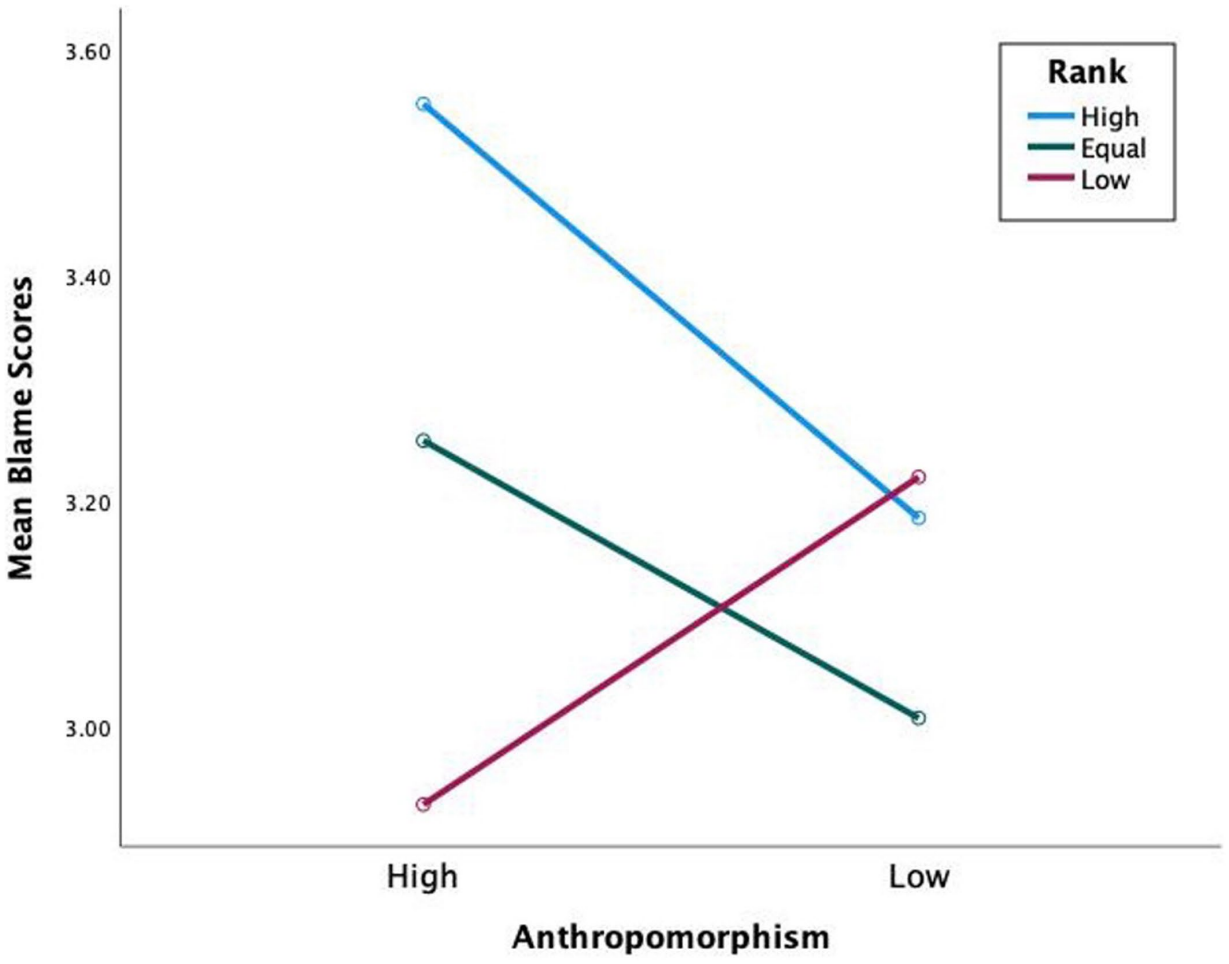
Mean blame scores for blame apportioning by anthropomorphism and rank.

**Figure 4:**
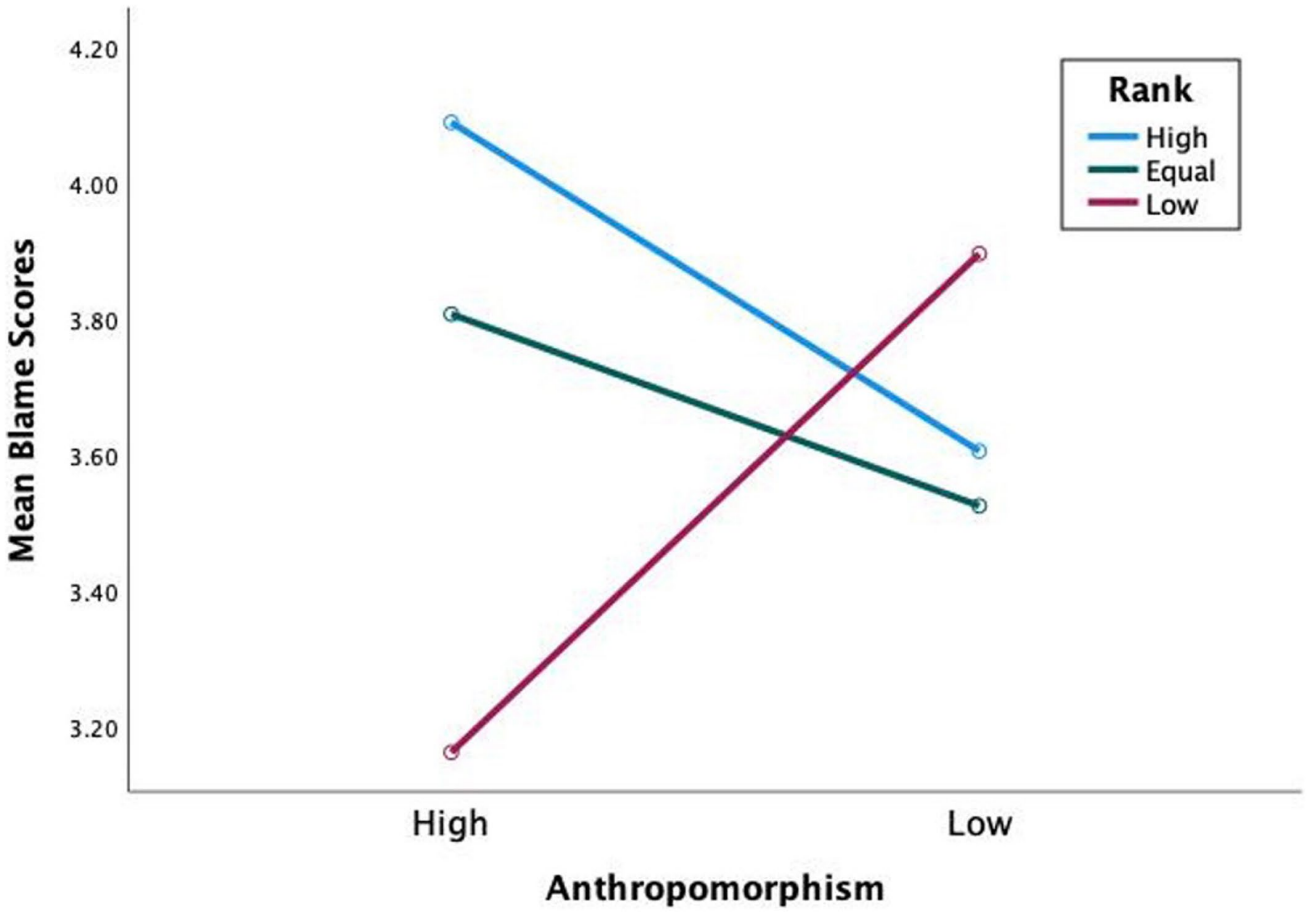
Mean blame scores for blame apportioning by anthropomorphism, rank during hard missions.

Hypothesis 1 states that participants apportion more blame to high-rank human-like teammates than low-rank human-like teammates. Sidak *post hoc* comparisons revealed a significant difference of blame apportioned to high-rank human-like than low-rank human-like (*M*_Diff_ = 0.62, Sidak 95% CI [0.09, 1.15]) (see Figure 3). With task difficulty included in the model (see Figure 4), a significant three-way interaction was found *F*(2,60) = 4.51, *p* = 0.015, η_p_^2^ = 0.13, with more blame apportioned during hard missions to high-rank human-like than low-rank human-like (*M*_Diff_ = 0.92, Sidak 95% CI [0.31, 1.55]), and equal-rank human-like than low-rank human-like (*M*_Diff_ = 0.65, Sidak 95% CI [0.05, 1.24]). The results support hypothesis 1.

Hypothesis 2 states that participants blame high-rank human-like teammates more than high-rank machine-like ones. Further, blame equal-rank and low-rank human-like less than equal-rank and low-rank machine-like. Sidak *post hoc* comparisons revealed no significant difference of blame apportioned between high-rank human-like and high-rank machine-like (*M*_Diff_ = 0.37, Sidak 95% CI [0.05, 0.78]), equal-rank human-like and equal-rank machine-like (*M*_Diff_ = 0.25, Sidak 95% CI [0.08, 0.57]), or low-rank human-like and low-rank machine-like (*M*_Diff_ = 0.29, Sidak 95% CI [0.09, 0.67]) (see Figure 3). However, remember the significant three-way interaction of anthropomorphism, rank, and task difficulty (see Figure 4). Sidak *post hoc* comparisons found evidence that when missions were hard, participants blamed low-rank human-like teammates significantly less than low-rank machine-like (*M*_Diff_ = 0.73, Sidak 95% CI [0.25, 1.22]), partially supporting hypothesis 2.

A measure of trust was inferred from the participant’s behaviour during the MAHVS simulation. Recommendations manually accepted by participants were considered a measure of trust (*N* = 1,688), whilst recommendations manually rejected or ignored, or when participants took manual control of the aircraft, were considered a measure of no trust (*N* = 6,507). Frequencies of independent variables can be found in Table 6. Binomial logistic regression was the most appropriate test to analyse trust data due to a binary dependent variable and an imbalance of manipulated variables. As such, assumption testing found significant Hosmer and Lemeshow tests for goodness of fit. Crosstabulation checks between independent variables showed balanced data for all groups except anthropomorphism/difficulty and rank/difficulty, with an unequal distribution of hard trials compared to easy trials. Therefore, any results and discussion surrounding trust and task difficulty should be interpreted cautiously.

**Table 6:** Frequencies of independent variables during the MAHVS simulation.

Variables									
	Teammate type		Rank			Decision cost		Difficulty	
*f*	Human-like	Machine-like	High	Equal	Low	Passengers	Supplies	Easy	Hard
*N*	3,992	4,203	3,231	2,729	2,235	3,770	4,425	2049	6,146
%	48.7	51.3	39.4	33.3	27.3	46	54	25	75

To investigate the likelihood that anthropomorphism, rank, decision cost, and task difficulty affect participants’ trust, binomial logistic regression was performed. Results were statistically significant, X^2^(11) = 298.39, *p* < 0.001. A summary of the results can be found in Table 7. The regression showed that machine-like teammates were 0.68 times less likely to be trusted than human-like teammates. Low-rank teammates were 1.56 times more likely to be trusted than high-rank teammates, and operating during hard missions was associated with 0.43 times less trust than operating during easy missions. The cost of decisions was not significant, although a three-way interaction with anthropomorphism and rank was *p* < 0.001. Participants trusted low-rank and equal-rank machine-like teammates 2.1 and 1.7 times more than high-rank human-like teammates when the decision cost was materials compared to humans. In addition, an interaction between anthropomorphism and rank was significant *p = 0*.004; however, comparing low and equal-rank machine-like teammates with high-rank human-like teammates failed to reach significance.

**Table 7:** Likelihood of predictors to influence trust.

						95% CI	
Predictors	β	*SE*	Wald	*p*	Odds ratio	*LL*	*UL*
Constant	−0.81	0.09	80.05	<0.001	0.45		
Anthropomorphism^1^	−0.38	0.09	16.34	<0.001	0.68	0.57	0.82
Rank			23.59	<0.001			
Rank^1^	0.44	0.10	20.91	<0.001	1.56	1.29	1.88
Rank^2^	0.07	0.09	0.51	0.473	1.07	0.89	1.28
Decision Cost^1^	0.00	0.07	0.00	0.995	1.00	0.87	1.15
Difficulty^1^	−0.85	0.07	153.37	<0.001	0.43	0.37	0.49
Anthro*Rank			10.83	0.004			
Anthro^1^* Rank^1^	−0.34	0.17	3.77	0.052	0.71	0.51	1.00
Anthro^1^* Rank^2^	0.27	0.16	2.70	0.101	1.31	0.95	1.80
Anthro*Cost*Rank			15.09	<0.001			
Anthro^1^*Cost^1^* Rank^1^	0.74	0.23	10.21	0.001	2.09	1.33	3.27
Anthro^1^*Cost^1^* Rank^2^	0.51	0.19	6.92	0.009	1.66	1.14	2.43
Anthro*Diff*Cost* Rank	−0.01	0.23	0.00	0.956	0.99	0.63	1.54
Anthro^1^*Diff^1^*Cost^1^* Rank^2^	−0.03	0.19	0.02	0.878	0.97	0.67	1.42

LL = Lower Limit; UL = Upper Limit. * Denotes interaction between predictors. Predictors and interactions are significant at *p* < 0.05. Anthropomorphism (Anthro) 0 = High, 1 = Low. Rank 0 = High, 1 = Low, 2 = Equal. Decision Cost (Cost) 0 = Human, 1 = Supplies. Difficulty (Diff) 0 = Easy, 1 = hard.

To decompose the interaction of anthropomorphism and rank and test our trust hypotheses, split files for anthropomorphism and rank were deemed necessary, with additional binomial logistic regressions.

**Figure 5:**
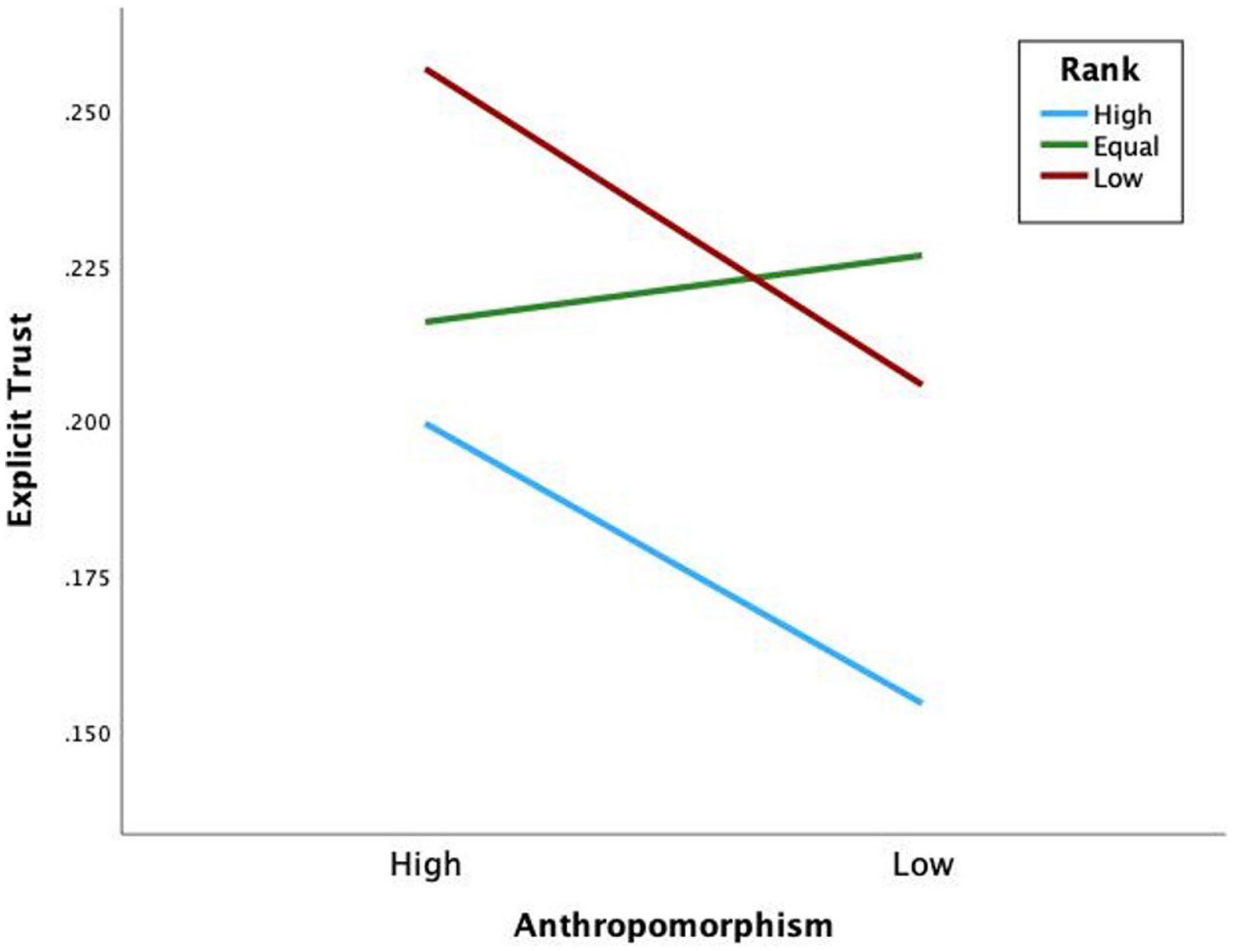
Explicit trust by anthropomorphism and rank.

Hypothesis 3 states that high-rank human-like teammates are trusted more than high-rank machine-like teammates. With the split file on for rank, the binomial logistic regression model was statistically significant for high-rank X^2^(3) = 84.59, *p* < 0.001, equal-rank X^2^(3) = 80.54, *p* < 0.001, and low-rank X^2^(4) = 99.90, *p* < 0.001. The regression showed that high-rank machine-like teammates were 0.69 times less likely to be trusted than high-rank human-like teammates, supporting hypothesis 3 (see Figure 5).

Hypothesis 4 states that high-rank human-like teammates are more likely to be trusted than low-rank human-like teammates. Hypothesis 5 states there will be no difference in trust between high-rank machine-like and low-rank machine-like. With the split file on for anthropomorphism, the binomial logistic regression model was statistically significant for human-like teammates X^2^(4) = 130.50, *p* < 0.001, and machine-like teammates X^2^(4) = 133.22, *p* < 0.001. Low-rank human-like were 1.53 times more likely trusted than high-rank human-like. Therefore, Hypothesis 4 was not supported. Between ranks for machine-like teammates, low-rank and equal-rank were 1.68 and 1.86 times more likely trusted than high-rank. Therefore, Hypothesis 5 was not supported.

### 3.2 Qualitative

All open-ended questions were analysed using Braun and Clarke’s (2006) six phases of thematic analysis. From the semantic content, the analysis focused on trust and blame apportioning using two themes. First, category-based, which investigates the effect of anthropomorphism and the status of artificial teammates on trust and blame apportioning. Second, person-based, which examines the impact of predictability and dependability.

The first category-based theme explores why artificial agents are trusted and blamed based on their level of anthropomorphism and status. When analysing why participants trust teammates the most, category-based responses such as “Felt like 3 stars and human attributes to have better judgement” and “More helpful than equivalent AI” were only reported when participants were teamed with human-like teammates. When analysing why participants trust teammates the least, category-based responses were only reported for low-rank teammates.

When analysing why participants blame teammates the most, category-based responses such as “Expected more,” “Expected more from 3-star agents,” and “Should have been programmed more effectively” were only found for high-rank teammates. When analysing why participants blame teammates the least, category-based responses such as “least experienced + more likely to need skill development” and “I had to take the lead due to my ranking” were only found for low-rank teammates.

The second theme, person-based, explores why artificial agents are trusted and blamed based on their predictability and dependability during shared tasks. When analysing why participants trust teammates the most, person-based responses such as “gave good advice and created less confusion” and “programmed with experience to detect outcomes that may occur” were the only responses to machine-like teammates. When analysing why participants trust teammates the least, person-based responses were the only responses reported for equal-rank and higher-rank teammates.

When analysing why participants blame teammates the most, person-based responses such as “Created confusion” and “Inaccurate recommendations” were the only type of response for low-rank and equal-rank teammates. When analysing why participants blame teammates the least, person-based responses were the only responses reported for equal-rank and higher-rank teammates.

The results show that when considering whether artificial teammates are trustworthy, humans use category-based cues for equal rank and higher-ranked high-anthropomorphic agents only and only person-based cues for low-anthropomorphic agents, regardless of rank. Further, when considering whether artificial agents are to be blamed, humans only use category-based cues for high-rank artificial teammates and only person-based cues for equal or lower rank.

A summary of blame and trust hypotheses can be found in Table 8.

**Table 8:** Summary of blame and trust hypotheses.

Hypothesis	Test	Result	Originality
1. Participants apportion more blame to high-rank human-like teammates than low-rank human-like teammates	Binomial logistic regression	Supported	In accordance with Kaspar et al. (2016) previous findings in human-human interactions
2. Participants blame high-rank human-like teammates more than high-rank machine-like	Binomial logistic regression	Partially Supported	Newly proposed
3. High-rank human-like teammates are trusted more than high-rank machine-like teammates	One-way repeated measure ANOVA with Sidak *post hoc* comparisons.	Supported	Newly proposed
4. High-rank human-like teammates are more likely to be trusted than low-rank human-like teammates	One-way repeated measure ANOVA with Sidak *post hoc* comparisons.	Not Supported	Newly proposed
5. There will be no difference in trust between high-rank machine-like and low-rank machine-like teammates	One-way repeated measure ANOVA with Sidak *post hoc* comparisons.	Not Supported	Newly proposed

## 4 Discussion

This study investigated whether higher levels of anthropomorphism invoke the CASA paradigm, prompting humans to apply social rules to their interactions with computers similarly to human-human interactions. Our findings provide evidence that this occurs in human-agent interactions, with blame apportioning to superior and subordinate human-like agents like human employers and employees. In addition, we examined the effect power distance orientation has on users’ trust in autonomous agents and blame apportioning and whether similarity or complementarity on human individual differences that impact human-agent interaction is optimal. We found evidence that participants with low power distance orientation trust human-like subordinates more than superiors and blame them less.

Our research aimed to investigate whether higher levels of anthropomorphism invoke CASA effects. We hypothesised that if higher levels of anthropomorphism are more likely to invoke CASA effects, as suggested by Gambino et al. (2020), then priming social roles and hierarchies should prompt participants to apportion blame between superior and subordinate human-like teammates similarly to how they apportion blame in human vignettes demonstrated by Kaspar et al. (2016), where an employer receives significantly more blame for failures than an employee. We found evidence to support this. Further, when comparing blame apportioned between high-rank and low-rank computer-like teammates, there was no significant difference; in fact, participants blamed low-rank machine-like more than high-rank machine-like.

When analysing participant’s reasons for why an artificial teammate was most to blame, category-based responses such as “Expected more from 3-star agents” were only found for high-rank teammates, and person-based responses such as “Inaccurate recommendations” were the only type given to lower and equal-rank teammates. Considering this, we could argue that participants in our study blamed high-rank human-like teammates more than low-rank human-like teammates based on categorization, and low-rank machine-like more than high-rank machine-like based on predictability and dependability. This result should interest fellow researchers Willemsen et al. (2018) and Kaspar et al. (2016), who argue that in human-human interactions, social roles and hierarchies such as supervisor and subordinate can significantly affect the attribution of praise and blame. Further, Lei and Rau (2021) found that when shared tasks between participants and a high-anthropomorphic robot fail, people exhibit self-serving biases when Nao acts as a supervisor but not as a peer or subordinate.

Our findings support the CASA paradigm (Nass et al., 1994) by demonstrating that higher levels of anthropomorphism invoke CASA effects (Gambino et al., 2020). In addition, we seek to use this effect to explain conflicting findings from past research.

In their recent review of the HAT literature, Lyons et al. (2021) found robots are given less blame for failure relative to humans, citing Li et al. (2016) study as an example, where participants apportioned less blame to an autonomous car relative to a human driver when both are at fault for an accident. Not mentioned, however, was that participants blamed the manufacturer/software developer of the car and government more than the human driver, with the study researchers suggesting the autonomous vehicle may not be perceived to be a moral agent worthy of blame. Considering this, we argue that autonomous vehicles are void of anthropomorphism, which prompts participants to direct social rules like blame apportioning towards anthropomorphic entities such as manufacturers and software developers, similar to participants in Liu and Du’s (2021) study, who apportioned more blame to automation and designers presented dependently, than to humans.

Suppose higher levels of anthropomorphism invoke CASA effects, as Gambino et al. (2020) suggested. In that case, priming social roles and hierarchies should prompt participants to trust high-rank human-like teammates more than high-rank machine-like teammates. Our hypothesis was informed by Adams and Webb’s (2002) examination of trust in small military teams, with the authors suggesting a positive reputation is often conferred on higher-ranked individuals because of the authority granted to these leaders, with increased trust influenced by such categorization. We found evidence to support this. Further evidence of this effect was found when analysing responses to which teammates were most trustworthy and why, with category-based responses such as “This agent had human-like features and had a 3-star rating which led me to put more trust in its decisions,” only reported when participants were teamed with high-rank human-like teammates. For high-rank machine-like teammates, only person-based responses such as “programmed with experience” were reported, suggesting that they are only trusted based on their predictability and dependability.

We expected further evidence that high levels of anthropomorphism influence trust in a military context, with high-rank trust more than low-rank trust for human-like teammates, but no difference in trust associated with different ranks for machine-like teammates. However, the results went in the opposite direction. Low-rank human-like and low-rank machine-like teammates were trusted more than high-rank human-like and high-rank machine-like.

Adams and Webb (2002) suggest category-based trust can emerge before person-based trust, based on whether a person is predictable and dependable. Considering all AI teammates operated with the same level of competency regardless of rank, perhaps person-based trust influenced participants’ behaviour more than category-based trust during shared tasks. Support for this assumption was found when analysing the qualitative data. For example, one participant stated, ‘I started trusting based on the ranking but evolved to trust based on the situation’. Furthermore, another, when answering a question about which teammate they trusted most and why, “Agent 5 (high-rank human-like) based on stars, but as the game moved forward, I found Agent 1(low-rank human-like) more trustworthy”. Another explanation is the influence of power distance orientation.

Our second objective was to investigate power distance orientation’s effect on users’ trust in autonomous agents and blame apportioning. In cultures with high power distance, there is a belief that low-status individuals must accept instructions from high-status individuals (Hofstede, 1991). If individuals with a high-power distance orientation see automation as authoritative, they will quickly develop trust in automated recommendations (Chien, 2016). In the present study, the power distance orientation scale revealed participants’ low power distance orientation. We could argue that participants assigned a lower rank than their high-rank human-like and machine-like teammates ignored or rejected agent recommendations as they are less receptive to top-down orders. Interestingly, in a research study in the United Kingdom, a country low in power distance like Australia, Beton et al. (2017) found that when paired with a robot teammate of equal status during a team task, participants rated robots that adopted a follower collaboration strategy more trustworthy than a robot that took the lead.

Our results for blame apportioning suggest an influence of power distance orientation. Whilst higher levels of anthropomorphism invoked CASA effects, in low power distance countries like Australia, blame is placed on people higher up the hierarchy (Hofstede, 2001). Our findings support this, with significantly more blame apportioning to high-rank human-like teammates than low-rank human-like teammates. Interestingly, Hinds et al. (2004) found that participants felt less responsible when paired with robot supervisors than subordinates and peers but blamed robot supervisors significantly more. The study was conducted in the United States, which, according to Hofstede (2001), is a low power distance country like Australia.

Our third objective was to ascertain whether the similarity or complementarity of human difference variables that impact trust and blame apportioning is optimal. In a previous study of HAT in Australia, a low power distance country, researchers Sharifheravi et al. (2020) investigated the effect anthropomorphism has on trust in AI teammates during shared tasks, with more trust expected with high-anthropomorphic teammates than low-anthropomorphic teammates; however, this was disproven, with the researchers suggesting their human-like avatar may not have been anthropomorphic enough to confirm their hypothesis. Nevertheless, Sharifheravi et al. (2020) hypothesis was informed by previous studies of anthropomorphism and trust, including a study by Calhoun et al. (2019), who found that participants from the United States, another low-power distance country trusted an automated aid with higher levels of humanness more than lower levels of humanness to assist in insurgent identifications. To extend the findings of Calhoun et al. (2019) and address the limitations set out by Sharifheravi et al. (2020), we confirmed our manipulation of anthropomorphism. We found that participants from Australia trust human-like teammates more than machine-like teammates.

To invoke power-related effects, we introduced agent rank, manipulated as either subordinate, peer, or superior to participants. We found evidence that participants with low power distance orientation trust human-like subordinates more than superiors and blame them less. Our results suggest that similarities of human characteristics combined with a complementary lower-ranked status are optimal for increasing human teammates’ trust in AI teammates and a sense of responsibility for shared task failures. This finding should interest O’Neill et al. (2020), who suggested more research was needed to ascertain whether similarity or complementarity on individual differences that impact human-agent interaction is optimal.

### 4.1 Limitations

The present study is not without limitations. First, whilst the narrative surrounding rank in post-experiment questionnaires suggests that our rank manipulation was adequate, our check adapted from Lei and Rau’s (2021) study into the effect of relative status on responsibility attributions in human-robot collaboration found no evidence to support this. In the present study, we hypothesise that measuring participants’ perception of their teammate’s leadership quality after each mission captured perception based on performance instead of category. Future researchers investigating the effect of rank based on category would best check the manipulation before each mission. Second, our manipulation of different eye colours, hair colours and hairstyles of our human-like avatars may have, independently or combined with the rank insignia, affected how participants assigned blame and trust beyond the rank manipulation. Future researchers investigating rank based on category should control such confounding variables in anthropomorphic manipulations. Last, our manipulation of anthropomorphism for the artificial teammates included facial and voice features, which were likely perceived as male. Future researchers investigating the effect of anthropomorphism could replicate our study using facial and voice features likely perceived as female to examine whether our findings are gender specific or not.

## 5 Conclusion

McNeese et al. (2018) predict that in the future, autonomous agents could replace human teammates as pilots, navigators, or photographers and conduct surveillance whilst negotiating enemy activity. Suppose humans are expected to complete shared tasks with autonomous agents in HAT. In that case, human factors that influence trust and shared accountability must be considered when designing artificial agents and creating policies for shared task failures. Our results suggest that humans trust human-like teammates more than machine-like teammates and trust higher-rank teammates less than lower-rank teammates. In addition, when apportioning blame, humans blame higher-rank human-like teammates more than lower-rank human-like teammates. In sum, when humans are teamed with artificial teammates of similar human characteristics, and a complementary lower-ranked status, a sense of responsibility for shared task failures and trust in artificial teammates is optimal.

## Data availability statement

The original contributions presented in the study are included in the article/Supplementary material, further inquiries can be directed to the corresponding author.

## Ethics statement

The studies involving humans were approved by the Human Research Ethics Committee of Western Sydney University. The studies were conducted in accordance with the local legislation and institutional requirements. The participants provided their written informed consent to participate in this study.

## Author contributions

JG: Writing – review & editing, Writing – original draft, Visualization, Project administration, Methodology, Investigation, Formal analysis, Conceptualization. CS: Writing – review & editing, Supervision, Software, Resources.
