# Supplementary material for: Low-rank human-like agents are trusted more and blamed less in human-autonomy teaming
Source: Front Artif Intell. 2024 Apr 29;7:1273350. doi: 10.3389/frai.2024.1273350 (PMC11089226; doi:10.3389/frai.2024.1273350)
Supplement: Supplementary file 1 [file Data_Sheet_1.docx]

**Appendix**

**Cover Story and Scales used to Measure Blame, Human-Likeness and Power Distance Orientation**

Thank you for your participation. Please refer to Figure 1.

In today’s experiment, you have been tasked with guiding numerous aircraft to safety (indicated by the runway in Figure 1).

The aircraft will automatically land; however, your mission is to avoid aircraft collisions (highlighted by the red dots) and enemy fire within the danger zone (indicated by the red rectangle).

Missions will vary in the number of supplies carried by drones and the number of passengers carried by passenger jets, indicated by the number above the aircraft (in Figure 1-2, drones are carrying 21kg & 18kg of supplies, and three jets are carrying 27, 17 & 13 passengers).

Your missions will vary in task difficulty, either easy or hard, with more aircraft in hard missions than easy missions.

During the mission, an AI teammate will offer routing recommendations indicated by green arrows over the aircraft, which you can accept, reject, or ignore. You can accept recommendations by clicking on the arrow, which will change the aircraft's flight path, reject them by clicking on the red cross, ignore them, or manually control the aircraft by clicking directly.

Please refer to Figure 2

AI teammates are not perfect, and sometimes, you might wish to ignore recommendations and manually control the aircraft. To do this, simply click on the aircraft and either up or down on the green arrow, which will change the aircraft's flight path in that direction. By changing flight paths, the aircraft will take longer to land and impact your overall score.

Please refer to Figure 3

At the end of each mission, you will receive a score for the number of supplies or passengers safely landed (indicated by the payload landed in figure 3) and the number of seconds left remaining from a countdown clock of 40sec (shown by the time: figure 3)

Missions last for 40 seconds, so accepting, rejecting, ignoring recommendations, or manually controlling the aircraft should only be done to avoid collisions or the danger zone. Remember, aircraft will automatically land unless they crash.

Please refer to Figure 4

Today, you will be paired with six different artificial teammates in random order and complete four missions with each. Rank is displayed on each teammate designated by the black stars.

After completing practice missions with different artificial teammates, you will be assigned a rank of either 1 star, 2 star, or 3 stars.

Before and after each mission, your artificial teammate will be displayed.

After each mission, you will be asked questions about your team’s performance.

Let us know any questions about the experiment before you start. Good Luck

**Front of Sheet given to participants for reference during a cover story**


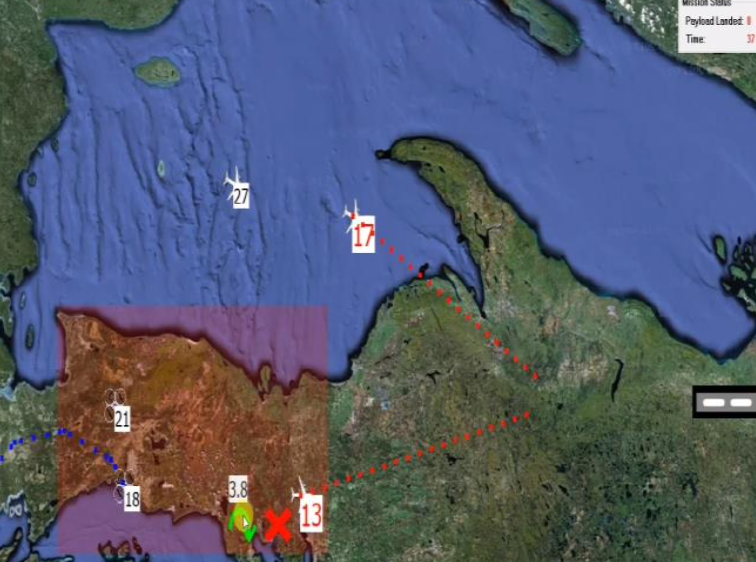


**Figure 1**


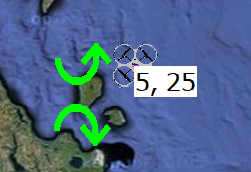

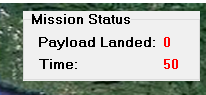


**40**

**30**

**Figure 2 Figure 3**

| 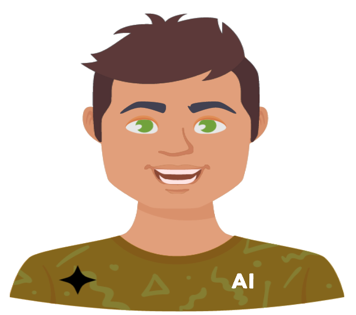 Agent 1 | 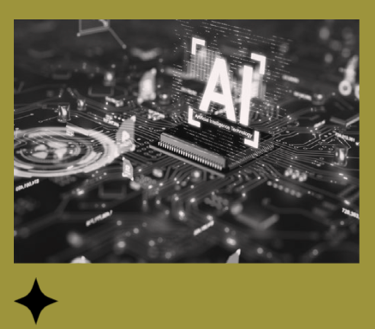 Agent 2 |  |
| --- | --- | --- |
| 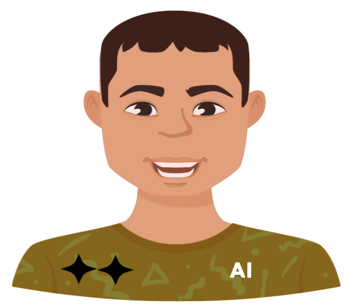 Agent 3 | 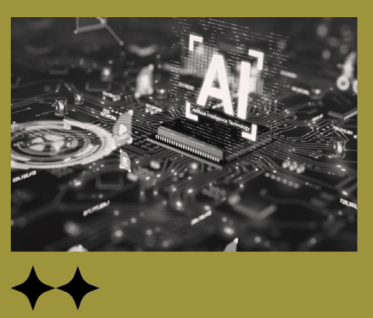 Agent 4 | |
| 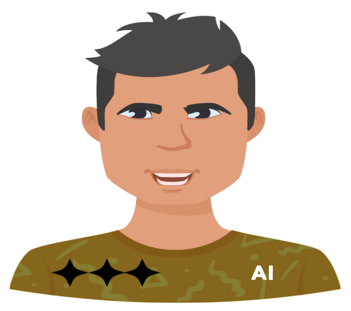  Agent 5 | 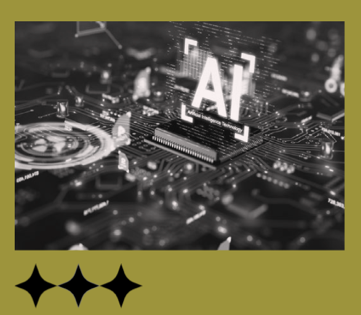  Agent 6 | |

**Back of the Sheet was given to participants for reference during the cover story**

**Figure 4**

**Table B1**

*Items used to measure participant’s attribution of blame after each trial*

| Scales and items | Cronbach’s 𝛼 |
| --- | --- |
| Attribution of Blame | .85 |
| I hold my partner responsible for any errors that we made on this task. |  |
| My partner is to blame for most of the problems we encountered in accomplishing this task. |  |

**Table B2**

*Items used to measure participant’s perception of teammate’s human likeness*

| Scale and items | Cronbach’s 𝛼 |
| --- | --- |
| Human Likeness | .80 |
| To what extent do the robots |  |
| Have human-like attributes? |  |
| Look like a machine or mechanical device? ª |  |
| Have characteristics that you would expect of a human? |  |
| Look like a person? |  |
| Have machine-like attributes? ª |  |
| Act like a person? |  |
| Act like a machine? ª |  |

ª Item was reverse scored.

**Table B3**

*Items used to measure participant’s power distance orientation*

| Scale and items | Cronbach’s 𝛼 |
| --- | --- |
| Power Distance Orientation | .762 |
| In most situations, managers should make decisions without consulting their subordinates. |  |
| Once a top-level executive makes a decision, people working for the company should not question it. |  |
| In work-related matters, managers have the right to expect obedience from their subordinates. |  |
| Employees should not express disagreement to their managers. |  |
| Managers should be able to make the right decisions without consulting others. |  |
| Employees should not disagree with management decisions. |  |
| It is better for people to not question the decisions of those in authority. |  |

**Supplementary Material for Qualitative Analysis**

**Open Ended Questions and Answers from post experiment questionnaire**

**Which teammate did you trust the most and why?**

Id: 1 Agent 5- “Highest ranking human, feel like I achieved best results with agent 5”

Id: 2 Agent 3- “Agent 3 seemed to be considerate of moves I would likely make and was more helpful”

Id: 3 Agent 5- “Because he helped out in more successful ways”

Id: 4 Agent 3– “Had more confidence in decisions”

Id: 5 Agent 3– “Didn’t make as many decisions or mistakes”

Id: 6 Agent 3– “Seemed to be a bit more helpful with leading the drone/plane to the runway”

Id: 7 Agent 3– “Good multi-tasking”

Id: 8 Agent – “Did not pay attention. Sorry”

Id: 9 Agent – “Don’t remember”

Id: 10 Agent 3– “Assisted with the most accurate flight path and passengers landed on the runway.”

Id: 11 Agent 5– “Made some good decisions and allowed me to control”

Id: 12 Agent – “Based on stars but as the game moved forward found agent 1 more trustworthy”

Id: 13 Agent 1– “The reason why agent 1 is because he gave more directions”

Id: 14 Agent 6– “Seem to give good advice and create less confusion”

Id: 15 Agent 4– “Most consistent in when it made a decision. I knew what to expect”

Id: 16 Agent 3– “More ‘humanistic’ than equivalent AI agent”

Id: 17 Agent 1– “Most decisions led to success in my memory”

Id: 18 Agent 4– “No people/materials were lost with Agent 4. In general, it seemed AI agents to be more trustworthy in respect to any errors (human agents more errors)”

Id: 19 Agent None– “At the start, I trusted the higher ranked but as it went on saw all were capable and uncapable”

Id: 20 Agent 3- “The recommendations provided were more accurate than the other agents”

Id: 21 Agent 5– “This agent had human-like features and had a 3-star rating which led me to put more trust in its decisions”

Id: 22 Agent 5– “Trusted Agent 5 the most. Felt like 3 stars and human attributes to have better judgement”

Id: 23 Agent 3– “Was more rounds appeared easier to make recommendations”

Id: 25 Agent 5– “As I trust him the most make right call if I could not”

Id: 26 Agent 5– “Looks older, more experienced. Hair colour looks grey. 3 stars more senior”

Id: 27 Agent 5 & 6– “Agent 5 More experienced-looks like he’s knowledgeable. Serious looking, gets the task done, more relatable. Agent 6 It’s programmed with experience to detect outcomes that may occur.”

Id: 28 Agent 3– “Because he seemed the most nice/neutral when he said he was here to help”

Id: 29 Agent 5– “Due to his 3 stars/experience”

Id: 30 Agent 6– “We seemed to have least amount of crashes while still completing some missions”

Id: 31 Agent 4– “Least amount of collisions”

Id: 28 Agent 5– “Most stars”

**Which teammate did you trust the least and why?**

Id: 1 Agent 2- “Lowest ranking robot/machine. Feel like I achieved the worst results.”

Id: 2 Agent 5- “Moves were made quickly with little consideration to other ‘players’ me in the game”

Id: 3 Agent 2- “Because they didn’t help as much”

Id: 4 Agent 1– “Looked nice and trustworthy but was the opposite”

Id: 5 Agent 5– “Made to many decisions that were panicked and wrong”

Id: 6 Agent 5– “Seemed to be making all these changes causing the aircrafts to collide”

Id: 7 Agent 6– “Too many mistakes”

Id: 8 Agent – “Did not pay attention. Sorry”

Id: 9 Agent – “Don’t remember”

Id: 10 Agent 1– “Assistance with the appropriate flight path was incorrect most of the time and the most kills associated”

Id: 11 Agent 1– “Left things to me and didn’t help in last minute changes”

Id: 12 Agent 2– “Based on stars but also found agents 2, 4 & 6 unremarkable”

Id: 13 Agent 6– “The reason why agent 6 is because the machine gave no directions”

Id: 14 Agent 1– “Bad or no advice. Felt like they were confusing the situation and not helping”

Id: 15 Agent 3– “It made decisions for itself in some scenarios but not others. It frustrated me in that I was unsure if I was responsible for all work”

Id: 16 Agent 2– “Least ‘experienced’ AI agent, without human characteristics of reasoning/empathy etc”

Id: 17 Agent 5– “Small mistakes which had to be corrected or things crashed”

Id: 18 Agent 1– “I can’t remember if people/materials were lost with agent 1, however, the ‘human’ agents were less trustworthy, hence I chose the lowest ranking”

Id: 19 Agent – “I start trusting based on the ranking but evolved to trust based on the situation”

Id: 20 Agent 1-

Id: 21 Agent 1– “Had the lowest rating and as it looked more human-like features which made me think that it was inclined to make more errors than the ‘robot’ with the same rating”

Id: 22 Agent 1– “Least amount of experience and knowledge”

Id: 23 Agent 5– “Want to make all the moves resulting in crashes”

Id: 25 Agent 2– “As I thought let me down the most on one of my rounds were I needed his support”

Id: 26 Agent 2– “Least experienced machine agent”

Id: 27 Agent 1 & 2– “Felt that I needed to step up and take the lead. Looks inexperienced. Agent 2 Not as far advanced as Agent 6”

Id: 28 Agent 5– “He seemed pushy”

Id: 29 Agent 2– “Not as experienced as the other options. Less than agent 1 due to agent 1 talking before the games started”

Id: 30 Agent 2– “We seemed to have more moves that took planes off task and more crashes”

Id: 31 Agent 3– “AI recommendations lead to collisions or landed in red rectangle”

Id: 32 Agent 1– “”

**Which teammate did you blame the most and why?**

Id: 1 Agent 2- “I feel most errors occurred with agent 2”

Id: 2 Agent - “All were equal, very little blame on any player as they were playing their game”

Id: 3 Agent - “I didn’t really blame any teammates more than the other as it was just as much my fault”

Id: 4 Agent 5– “More frustrating, too many crashes”

Id: 5 Agent 6– “Making wrong or unnecessary turns”

Id: 6 Agent 5– “Too many collisions when they would make changes in the direction that the aircraft was heading”

Id: 7 Agent 6– “Mistakes could have been avoided”

Id: 8 Agent – “Did not pay attention. Sorry”

Id: 9 Agent – “Don’t remember”

Id: 10 Agent Myself– “Ultimately I was responsible for the decisions made as I was in control of the buttons and the final decision was mine”

Id: 11 Agent 3 & 2– “decisions to change to longer route”

Id: 12 Agent 5– “Found suggestions didn’t work”

Id: 13 Agent 5– “The reason why is because Agent 5 expected you to understand how to complete the task without direction”

Id: 14 Agent 1– “Created confusion”

Id: 15 Agent 3– “Inconsistent and no real assistance initially when that is where I would expect AI workmate to help the most”

Id: 16 Agent None– “I had control over decision making overall, so errors are my responsibility. Agent 6 if I had to pick, should have been programmed more effectively”

Id: 17 Agent 5– “Decisions led to crashes or shot down”

Id: 18 Agent 1– “From memory, Agent 1 missed many opportunities to assist and save people/materials (in comparison to others-some which required no assistance on my end)”

Id: 19 Agent 5 & 6– “I expected more from the 3 star agents”

Id: 20 Agent 4- “Recommendations were not very accurate”

Id: 21 Agent 5– “As I trusted this agent the most, I made less manual direction changes, which than led to a high number of crashes”

Id: 22 Agent 4– “Some of the judgement calls still ended in crashes”

Id: 23 Agent Can’t remember– “”

Id: 25 Agent 3– “As kept sending my planes away when they didn’t need to be”

Id: 26 Agent – “Didn’t differentiate in my scoring according to which agent. Blame based on result of mission and amount of over-riding I did on AI suggestions”

Id: 27 Agent 5 & 6– “While I was re-directing planes we got shot at in the danger zone. ‘What was he doing?’ I had all these other planes + drones to look after. Agent 6 are you smarter than me?”

Id: 28 Agent 5– “Because he was more pushy I feel we did the worst”

Id: 29 Agent 1– “Made mistakes”

Id: 30 Agent – “”

Id: 31 Agent 5– “Did not give many recommendations”

Id: 32 Agent 1– “”

**Which teammate did you blame the least and why?**

Id: 1 Agent 5- “I blame agent 5 least, I feel agent 5 was most helpful”

Id: 2 Agent 3- “Agent 3 appeared to be considering my moves and gave time to respond”

Id: 3 Agent - “I didn’t really blame any teammates more than the other as it was just as much my fault”

Id: 4 Agent 3– “More success and less crashes”

Id: 5 Agent 3– “Seemed less erratic, didn’t make as many wrong decisions”

Id: 6 Agent 4– “I think. More helpful in suggestions when to change the aircrafts travel direction. More successful missions”

Id: 7 Agent 2– “Good directions”

Id: 8 Agent – “Did not pay attention. Sorry”

Id: 9 Agent – “Don’t remember”

Id: 10 Agent 3– “Assisted with the most appropriate action and had the better results”

Id: 11 Agent 5– “Made last minute changes but allowed y changes too”

Id: 12 Agent 2– “Don’t recall any problems”

Id: 13 Agent 1 or 3– “The reason why is because Agent 1 gave you heaps of help and Agent 3 gave you direction and help when need”

Id: 14 Agent 5– “Felt like they weren’t perfect but didn’t create confusion”

Id: 15 Agent 4– “Most consistent and the scenarios seemed to run more smoothly”

Id: 16 Agent 1– “least experienced + more likely to need skill development”

Id: 17 Agent 1– “Most success”

Id: 18 Agent 6– “From memory, the least amount of people/materials were lost with Agent 6- hence, there was no errors considering blame or responsibility”

Id: 19 Agent Not sure– “At the start I expected/relied on 3 stars more but overall I had the final say. When it was busy and there was problems if I didn’t instruct the agent to make that choice it was still my choice to trust the agent”

Id: 20 Agent 3- “Accurate recommendations”

Id: 21 Agent 2– “I made more manual changes with this agent, so I relied less on its recommendations. This meant we made more successful landings”

Id: 22 Agent 5– “Because it felt like a team and we equally contributed to the decision making in the experiment”

Id: 23 Agent 1 or 2– “Felt I had more input and faith in decisions that were made to reduce the amount of casualties”

Id: 25 Agent 5– “Was solid teammate made right calls at right times”

Id: 26 Agent – “Couldn’t say which one”

Id: 27 Agent 1 & 2– “I had to take the lead due to my ranking”

Id: 28 Agent 3– “Because we did the best”

Id: 29 Agent 4– “Made the least mistakes from memory”

Id: 30 Agent – “”

Id: 31 Agent 4– “Recommendations were accurate and helped me achieve my score”

Id: 32 Agent 6– “”

**Appendix C**

**Supplementary Material for Manipulation Checks and Power Distance Orientation Analysis**

**Table C1**

*Means and Standard Deviations from human-likeness scale*

|  |  | *M* | *N* | *SD* |
| --- | --- | --- | --- | --- |
| Pair 1 | Human-Like | 4.76 | 31 | 1.32 |
|  | Machine-Like | 2.03 | 31 | .94 |

**Table C2**

*Correlations between human-like and machine-like responses to human-likeness scale*

|  |  | *N* | *r* | *p* |
| --- | --- | --- | --- | --- |
| Pair 1 | Human-Like  Machine-Like | 31 | -.25 | .179 |

**Table C3**

*Results for paired samples t-test from human-likeness scale*

| Paired Differences | | | | | | | | | | | |
| --- | --- | --- | --- | --- | --- | --- | --- | --- | --- | --- | --- |
|  |  | *M* | *SD* | *SE* | 95% CI | | | *t* | df | *p* | |
|  |  |  |  |  | *LL* | *UL* | |  |  |  |  |
| Pair 1 | Human-Like  Machine-Like | 2.75 | 1.80 | .24 | 2.09 | | 3.41 | 8.50 | 30 | <.001 | |

*Note. LL* = Lower Limit*; UL* = Upper Limit

**Table C4**

*Means and Standard Deviations from follower leader question*

|  | *M* | *N* | *SD* |
| --- | --- | --- | --- |
| High-Rank Human-Like | 3.89 | 31 | 1.06 |
| Equal-Rank Human-Like | 3.75 | 31 | 1.10 |
| Low-Rank Human-Like | 3.80 | 31 | 1.09 |
| High-Rank Machine-Like | 3.89 | 31 | 1.39 |
| Equal-Rank Machine-Like | 3.95 | 31 | .96 |
| Low-Rank Machine-Like | 3.77 | 31 | 1.12 |

**Table C5**

*One-way repeated measures ANOVA results for follower leader responses*

| Predictor | *SS* | *df* | *MS* | *F* | *p* | η_p_^2^ |
| --- | --- | --- | --- | --- | --- | --- |
| Follower Leader | .96 | 3.67 | .26 | .21 | .921 | .01 |

*Note.* Greenhouse-Geisser correction used for sphericity violation

**Table C6**

*Means and Standard Deviations from power distance orientation scale*

|  | *M* | *N* | *SD* |
| --- | --- | --- | --- |
| Power Distance Orientation Scale | 2.91 | 31 | .96 |

**Appendix D**

**Supplementary Materials for Blame Analysis**

**Table D1**

*One-way repeated measures ANOVA results for blame*

| Predictor | *SS* | *df* | *MS* | *F* | *p* | η_p_^2^ |
| --- | --- | --- | --- | --- | --- | --- |
| Anthropomorphism | 2.15 | 1 | 2.15 | .86 | .362 | .03 |
| Rank | 11.99 | 2 | 5.99 | 1.86 | .165 | .06 |
| Cost | 3.50 | 1 | 3.50 | 2.05 | .163 | .06 |
| Difficulty | 177.11 | 1 | 177.11 | 33.56 | <.001 | .53 |
| Anthropomorphism*Rank | 15.17 | 2 | 7.59 | 4.02 | .023 | .12 |
| Anthropomorphism *Cost | 1.94 | 1 | 1.94 | 1.12 | .299 | .04 |
| Rank*Cost | 6.88 | 2 | 3.44 | 2.34 | .105 | .07 |
| Anthropomorphism*Rank*Cost | 7.94 | 2 | 3.97 | 2.29 | .110 | .07 |
| Anthropomorphism * Difficulty | 1.74 | 1 | 1.74 | .98 | .329 | .03 |
| Rank*Difficulty | .86 | 2 | .44 | .16 | .850 | .01 |
| Anthropomorphism*Rank* Difficulty | 11.39 | 2 | 5.69 | 4.51 | .015 | .13 |
| Cost*Difficulty | .84 | 1 | .84 | .63 | .435 | .02 |
| Anthropomorphism*Cost*Difficulty | 1.05 | 1 | 1.05 | .64 | .429 | .02 |
| Rank*Cost*Difficulty | 12.48 | 2 | 6.24 | 4.07 | .022 | .12 |
| Anthropomorphism*Rank*Cost*Difficulty | 7.80 | 2 | 3.90 | 2.63 | .081 | .08 |

*Note. LL* = Lower Limit*; UL* = Upper Limit. *** Denotes interaction between predictors. Predictors and interactions are significant at *p* <.05.

**Table D2**

*Difficulty Means*

|  |  |  | 95% CI | |
| --- | --- | --- | --- | --- |
| Difficulty | *M* | *SE* | *LL* | *UL* |
| Easy | 2.70 | .22 | 2.26 | 3.15 |
| Hard | 3.68 | .26 | 3.12 | 4.21 |

*Note. LL* = Lower Limit*; UL* = Upper Limit

**Table D3**

*Difficulty Pairwise Comparisons*

|  |  |  |  |  | 95% CI | |
| --- | --- | --- | --- | --- | --- | --- |
| Difficulty | | *M_Diff_* | *SE* | *p* | *LL* | *UL* |
| Easy | Hard | -.98 | .17 | <.001 | -1.32 | -.63 |

*Note. LL* = Lower Limit*; UL* = Upper Limit

**Table D4**

*Anthropomorphism*Rank Means*

|  |  |  |  | 95% CI | |
| --- | --- | --- | --- | --- | --- |
| Anthropomorphism | Rank | *M* | *SE* | *LL* | *UL* |
| Human-Like | High | 3.55 | .28 | 2.99 | 4.12 |
|  | Equal | 3.25 | .25 | 2.74 | 3.77 |
|  | Low | 2.93 | .29 | 2.35 | 3.51 |
| Machine-Like | High | 3.19 | .26 | 2.66 | 3.71 |
|  | Equal | 3.01 | .23 | 2.53 | 3.48 |
|  | Low | 3.22 | .25 | 2.72 | 3.73 |

*Note. LL* = Lower Limit*; UL* = Upper Limit

**Table D5**

*Anthropomorphism*Rank Pairwise Comparisons*

|  |  |  |  |  |  | 95% CI | | |
| --- | --- | --- | --- | --- | --- | --- | --- | --- |
| Anthropomorphism | Rank | Rank | *M_Diff_* | *SE* | *p* | *LL* | *UL* | |
| Human-Like | High | Equal | .30 | .18 | .11 | -.07 | .67 | |
|  |  | Low | .62 | .21 | .006 | .19 | 1.05 | |
|  | Equal | Low | .32 | .21 | .13 | -.10 | | .74 |
| Machine-Like | High | Equal | .18 | .20 | .39 | -.24 | .60 | |
|  |  | Low | -.04 | .24 | .88 | -.52 | .44 | |
|  | Equal | Low | -.21 | .18 | .25 | -.58 | .15 | |

*Note. LL* = Lower Limit*; UL* = Upper Limit

**Table D6**

*Anthropomorphism*Rank*Difficulty Means*

|  |  |  |  |  | 95% CI | |
| --- | --- | --- | --- | --- | --- | --- |
| Anthropomorphism | Rank | Difficulty | *M* | *SE* | *LL* | *UL* |
| Human-Like | High | Easy | 3.02 | .32 | 2.36 | 3.67 |
|  |  | Hard | 4.10 | .31 | 3.46 | 4.71 |
|  | Equal | Easy | 2.70 | .26 | 2.16 | 3.24 |
|  |  | Hard | 3.81 | .30 | 3.19 | 4.42 |
|  | Low | Easy | 2.70 | .28 | 2.13 | 3.28 |
|  |  | Hard | 3.16 | .36 | 2.43 | 3.89 |
| Machine-Like | High | Easy | 2.77 | .31 | 2.13 | 3.41 |
|  |  | Hard | 3.61 | .30 | 3.00 | 4.21 |
|  | Equal | Easy | 2.50 | .23 | 2.02 | 2.97 |
|  |  | Hard | 3.52 | .30 | 2.92 | 4.13 |
|  | Low | Easy | 2.55 | .25 | 2.04 | 3.06 |
|  |  | Hard | 3.90 | .31 | 3.27 | 4.52 |

*Note. LL* = Lower Limit*; UL* = Upper Limit

**Table D7**

*Anthropomorphism*Rank*Difficulty Pairwise Comparisons*

|  | | | | | | | 95% CI | |
| --- | --- | --- | --- | --- | --- | --- | --- | --- |
| Anthropomorphism | Rank | Difficulty | | *M_Diff_* | *SE* | *p* | *LL* | *UL* |
| Human-Like | High | Easy | Hard | -1.07 | .30 | <.001 | -1.66 | 3.67 |
|  | Equal | Easy | Hard | -1.11 | .27 | <.001 | -1.65 | -.56 |
|  | Low | Easy | Hard | -.46 | .30 | .138 | -1.01 | .16 |
| Machine-Like | High | Easy | Hard | -.84 | .32 | .014 | -1.50 | -.18 |
|  | Equal | Easy | Hard | -1.03 | .26 | <.001 | -1.57 | -.49 |
|  | Low | Easy | Hard | -1.35 | .26 | <.001 | -1.87 | -.83 |

*Note. LL* = Lower Limit*; UL* = Upper Limit

**Table D8**

|  |  |  |  |  |  |  | 95% CI | |
| --- | --- | --- | --- | --- | --- | --- | --- | --- |
| Anthropomorphism | Difficulty | Rank | Rank | *M_Diff_* | *SE* | *p* | *LL* | *UL* |
| Human-Like | Easy | High | Equal | .32 | .23 | .184 | -.16 | .79 |
|  |  |  | Low | .32 | .30 | .302 | -.30 | .93 |
|  |  | Equal | Low | .00 | .28 | 1.00 | -.58 | .58 |
|  | Hard | High | Equal | .28 | .25 | .27 | -.23 | .80 |
|  |  |  | Low | .93 | .25 | <.001 | .43 | 1.43 |
|  |  | Equal | Low | .65 | .29 | .035 | .05 | 1.24 |
| Machine-Like | Easy | High | Equal | .27 | .30 | .36 | -.33 | .88 |
|  |  |  | Low | .22 | .27 | .43 | -.33 | .77 |
|  |  | Equal | Low | -.06 | .24 | .81 | -.54 | .42 |
|  | Hard | High | Equal | .08 | .24 | .74 | -.40 | .56 |
|  |  |  | Low | -.29 | .33 | .38 | -.95 | .37 |
|  |  | Equal | Low | -.37 | .27 | .18 | -.92 | .18 |

*Note. LL* = Lower Limit*; UL* = Upper Limit

**Table D9**

|  |  |  |  |  |  |  | 95% CI | |
| --- | --- | --- | --- | --- | --- | --- | --- | --- |
| Rank | Difficulty | Anthropomorphism | | *M_Diff_* | *SE* | *p* | *LL* | *UL* |
| High | Easy | Human-like | Machine-like | -.25 | .19 | .194 | -.63 | .13 |
|  | Hard | Human-like | Machine-like | -.48 | .31 | .129 | -1.12 | .15 |
| Equal | Easy | Human-like | Machine-like | -.21 | .23 | .362 | -.67 | .25 |
|  | Hard | Human-like | Machine-like | -.28 | .20 | .162 | -.69 | .12 |
| Low | Easy | Human-like | Machine-like | -.15 | .25 | .544 | -.66 | .36 |
|  | Hard | Human-like | Machine-like | .73 | .24 | .005 | .25 | 1.22 |

*Note. LL* = Lower Limit*; UL* = Upper Limit

**Appendix E**

**Supplementary Material for Trust Analysis**

**Table E1**

*Likelihood of predictors to influence trust*

|  |  | |  |  |  |  | 95% CI | |
| --- | --- | --- | --- | --- | --- | --- | --- | --- |
| Predictors | | β | *SE* | Wald | *p* | Odds Ratio | *LL* | *UL* |
| Constant | | -.81 | .09 | 80.05 | <.001 | .45 |  |  |
| Anthropomorphism^1^ | | -.17 | .06 | 9.64 | .002 | .84 | .75 | .94 |
| Rank | |  |  | 47.41 | <.001 |  |  |  |
| Rank^1^ | | .46 | .07 | 43.51 | <.001 | 1.59 | 1.38 | 1.82 |
| Rank^2^ | | .33 | .07 | 24.15 | <.001 | 1.39 | 1.22 | 1.58 |
| Decision Cost^1^ | | .19 | .06 | 10.44 | .001 | 1.21 | 1.08 | 1.35 |
| Difficulty^1^ | | -.81 | .06 | 177.52 | <.001 | .44 | .39 | .50 |
| Anthro*Rank | |  |  | 10.83 | .004 |  |  |  |
| Anthro^1^* Rank^1^ | | -.34 | .17 | 3.77 | .052 | .71 | .51 | 1.00 |
| Anthro^1^* Rank^2^ | | .27 | .16 | 2.70 | .101 | 1.31 | .95 | 1.80 |
| Anthro*Cost*Rank | |  |  | 15.09 | <.001 |  |  |  |
| Anthro^1^*Cost^1^* Rank^1^ | | .74 | .23 | 10.21 | .001 | 2.09 | 1.33 | 3.27 |
| Anthro^1^*Cost^1^* Rank^2^ | | .51 | .19 | 6.92 | .009 | 1.66 | 1.14 | 2.43 |
| Anthro*Diff*Cost* Rank | | -.01 | .23 | .00 | .956 | .99 | .63 | 1.54 |
| Anthro^1^*Diff^1^*Cost^1^* Rank^2^ | | -.03 | .19 | .02 | .878 | .97 | .67 | 1.42 |

*Note. LL* = Lower Limit*; UL* = Upper Limit. *** Denotes interaction between predictors. Predictors and interactions are significant at *p* <.05.
Anthropomorphism (Anthro) 0 = High, 1 = Low. Rank 0 = High, 1 = Low, 2 = Equal. Decision Cost (Cost) 0 = Human, 1 = Supplies. Difficulty (Diff) 0 = Easy, 1 = hard

**Table E2**

*Likelihood of predictors to influence trust with split file on for rank*

|  |  |  | |  |  |  |  | 95% CI | |
| --- | --- | --- | --- | --- | --- | --- | --- | --- | --- |
| Rank | Predictors | | β | *SE* | Wald | *p* | Odds Ratio | *LL* | *UL* |
| High | Constant | | -.74 | .11 | 45.87 | <.001 | .48 |  |  |
|  | Anthropomorphism^1^ | | -.37 | .10 | 14.85 | <.001 | .69 | .58 | .84 |
|  | Decision Cost^1^ | | -.13 | .10 | 1.62 | .203 | .882 | .73 | 1.07 |
|  | Difficulty^1^ | | -.85 | .10 | 73.85 | <.001 | .43 | .35 | .52 |
| Equal | Constant | | -1.09 | .12 | 79.70 | <.001 | .34 |  |  |
|  | Anthropomorphism^1^ | | .17 | .10 | 3.28 | .070 | 1.19 | .99 | 1.43 |
|  | Decision Cost^1^ | | .40 | .10 | 16.19 | <.001 | 1.50 | 1.23 | 1.82 |
|  | Difficulty^1^ | | -.69 | .10 | 44.08 | <.001 | .50 | .41 | .62 |
| Low | Constant | | -.44 | .14 | 9.67 | .002 | .64 |  |  |
|  | Anthropomorphism^1^ | | -.29 | .11 | 7.80 | .005 | .75 | .61 | .92 |
|  | Decision Cost^1^ | | .37 | .11 | 12.05 | <.001 | 1.45 | 1.18 | 1.79 |
|  | Difficulty^1^ | | -1.06 | .12 | 76.98 | <.001 | .35 | .28 | .44 |

*Note. LL* = Lower Limit*; UL* = Upper Limit
Anthropomorphism 0 = High, 1 = Low. Decision Cost 0 = Human, 1 = Supplies. Difficulty 0 = Easy, 1 = hard.

**Table E3**

*Likelihood of predictors to influence trust with split file on for anthropomorphism*

|  |  |  | |  |  |  |  | 95% CI | |
| --- | --- | --- | --- | --- | --- | --- | --- | --- | --- |
| Anthropomorphism | Predictors | | β | *SE* | Wald | *p* | Odds Ratio | *LL* | *UL* |
| Human-like | Constant | | -.86 | .10 | 73.61 | <.001 | .42 |  |  |
|  | Rank | |  |  | 22.20 | <.001 |  |  |  |
|  | Rank^1^ | | .43 | .10 | 19.21 | <.001 | 1.53 | 1.27 | 1.86 |
|  | Rank^2^ | | .05 | .09 | .28 | .595 | 1.05 | .86 | 1.26 |
|  | Decision Cost^1^ | | .14 | .08 | 2.30 | .086 | 1.15 | .98 | 1.35 |
|  | Difficulty^1^ | | -.88 | .09 | 104.54 | <.001 | .42 | .35 | .49 |
| Machine-like | Constant | | -1.38 | .10 | 175.06 | <.001 | .25 |  |  |
|  | Rank | |  |  | 46.20 | <.001 |  |  |  |
|  | Rank^1^ | | .52 | .10 | 25.45 | <.001 | 1.68 | 1.37 | 2.05 |
|  | Rank^2^ | | .62 | .10 | 41.91 | <.001 | 1.86 | 1.54 | 2.25 |
|  | Decision Cost^1^ | | .26 | .08 | 9.44 | .002 | 1.29 | 1.10 | 1.52 |
|  | Difficulty^1^ | | -.80 | .09 | 82.54 | <.001 | .45 | .38 | .54 |

*Note. LL* = Lower Limit*; UL* = Upper Limit
Rank 0 = High, 1 = Low, 2 = Equal. Decision Cost 0 = Human, 1 = Supplies. Difficulty 0 = Easy, 1 = hard.
